# Characterization and Application of a New β-Galactosidase Gal42 From Marine Bacterium *Bacillus* sp. BY02

**DOI:** 10.3389/fmicb.2021.742300

**Published:** 2021-10-25

**Authors:** Zihan Zhou, Ningning He, Qi Han, Songshen Liu, Ruikun Xue, Jianhua Hao, Shangyong Li

**Affiliations:** ^1^School of Basic Medicine, Qingdao University, Qingdao, China; ^2^Key Laboratory of Sustainable Development of Polar Fishery, Ministry of Agriculture and Rural Affairs, Yellow Sea Fisheries Research Institute, Chinese Academy of Fishery Sciences, Qingdao, China; ^3^Jiangsu Collaborative Innovation Center for Exploitation and Utilization of Marine Biological Resource, Lianyungang, China

**Keywords:** β-galactosidase, glycoside hydrolase family 42, *Bacillus* sp. BY02, lactose hydrolysis, zinc ion

## Abstract

β-Galactosidase plays an important role in medicine and dairy industry. In this study, a new glycoside hydrolase family 42 (GH42) β-galactosidase-encoding gene, *gal42*, was cloned from a newly isolated marine bacterium *Bacillus* sp. BY02 and expressed in *Escherichia coli*. Structural characterization indicated that the encoding β-galactosidase, Gal42, is a homotrimer in solution, and homology modeling indicated that it retains the zinc binding sites of the Cys cluster. The reaction activity of Gal42 was significantly increased by Zn^2+^ (229.6%) and other divalent metal ions (Mn^2+^, Mg^2+^, and Co^2+^), while its activity was inhibited by EDTA (53.9%). Meanwhile, the thermo-stability of the Gal42 was also significantly enhanced by 5 and 10 mM of zinc ion supplement, which suggested that the “Cys-Zn” motif played important roles in both structural stability and catalytic function. Furthermore, Gal42 showed effective lactose hydrolysis activity, which makes the enzyme hydrolyze the lactose in milk effectively. These properties make Gal42 a potential candidate in food technology.

## Introduction

Currently, lactose intolerance is one of the most common nutritional disorders, with 70% of the world’s population affected by it (Horner et al., 2011; Di Costanzo and Berni Canani, 2018). The undigested lactose is fermented by colonic bacteria to produce gas and other by-products, leading to bloating cramps and diarrhea (Silberman and Jin, 2019). Removal of lactose from milk by the conversion of lactose to D-glucose and D-galactose is of great value for lactose-intolerant people.

β-Galactosidase (EC 3.2.1.23), also referred to as lactase, is an important member of glycosyl hydrolase, which can hydrolyze *O*-glycosidic bonds of lactose by hydrolysis reaction, resulting in the production of glucose and galactose (Vera et al., 2017; Singh et al., 2019). Treating dairy products with β-galactosidase through pre-hydrolyzation that can reduce lactose concentration offers a promising solution (Li et al., 2020). Thus far, numerous β-galactosidases were purified, cloned, and characterized from bacteria (Mavromatis et al., 2010; Tian et al., 2013), fungi (Rico-Díaz et al., 2017), yeast (de Freitas et al., 2020), plants (Deng et al., 2019), and mammals (He et al., 2008). Among all sources, microbial β-galactosidases have drawn extensive attraction due to their high yields, high activity, and abundance (Oliveira et al., 2011). In our previous research, a new β-galactosidase gene (*gal2A*) was cloned from the marine bacterium *Alteromonas* sp. QD01 and can be expressed in *Escherichia coli* (Li et al., 2020). According to the specific features of the sequence and reaction mechanism, β-galactosidases are classified as glycoside hydrolase (GH) families 1, 2, 35, 39, 42, 59, 147, and 165 in carbohydrate-active enzyme families.^1^ Among them, GH families 1, 2, and 35 contain many different types of polysaccharide-degrading enzymes, which can degrade different substrates. However, most of the enzymes belonging to GH family 42 (GH42) are β-galactosidase. Structurally, the GH family 42 representatives consist of three domains (Maksimainen et al., 2012). The first domain is a catalytic domain containing (α/β) eight barrels and two glutamic acid residues. The two highly conserved residues in all GH42 β-galactosidases act as an acid/base catalyst and a nucleophilic site (Barrangou et al., 2009). The second domain involves the trimer formation. The third domain forms a small β-barrel, and the function of domain C is unknown. In many cases, the applications of GH42 β-galactosidases are limited by their low activity, low thermal stability, or high inhibition by reaction products (Panesar et al., 2010; Zhang et al., 2018).

In this study, a new β-galactosidase encoding gene, *gal42*, was cloned from marine bacterium *Bacillus* sp. BY02 and expressed in *E. coli*. The recombinant β-galactosidase, Gal42, is a potential candidate in the production of lactose-free foods.

## Materials and Methods

### Materials

The *E. coli* strains, BL21 (DE3), and expression vector, pET28a(+), were purchased from Takara (Dalian, China). *O*-Nitrophenol (ONP) and *o*-nitrophenyl-β-D-galactopyranoside (ONPG) were purchased from Sangon Biotech (Shanghai, China). Lactose, glucose, and galactose used for this study were supplied by Solarbio (Beijing, China). The thin-layer chromatography (TLC) silica gel plates were purchased from Merck (Darmstadt, Germany). All other materials used were of the analytical degree.

### Isolation and Identification of *Bacillus* sp. BY02

The seawater samples were isolated and obtained from the surface of Yellow Sea sediment (depth 20 m, 120.16° E 35.01° N). They were diluted to 10 times then spread and cultured on Zobell 2216E medium (5.0 g of peptone, 1.0 g of yeast extract, 0.01 g of iron phosphate, 16.0 g of agar, and 1,000 ml of natural seawater were placed in the dark for several weeks, and the pH was adjusted to 7.6–7.8). A few strains that were positive were isolated after incubation at 25°C for 7 days. In order to identify which one can produce β-galactosidase, the selective medium [1% (w/v) peptone, 1% (w/v) yeast extract, 0.5% (w/v) lactose, 2% (w/v) NaCl, 0.004% (w/v) X-gal, and 1.5% (w/v) agar, pH 7.0] was used to culture and screen the strain. Among them, strain BY02 with high activity was collected and used for a further study. According to the 16S rRNA gene sequence analysis, strain BY02 was identified as a member of genus *Bacillus*. In order to evaluate the influence of lactose and glucose on the expression of β-galactosidase, strain BY02 was cultured on three kinds of medium (with 2% glucose, 2% lactose, 2% glucose, and lactose) for 3 days at 25°C.

### Sequence Analysis

The gene for β-galactosidase was amplified from the genome of *Bacillus* sp. BY02. The open reading frame (ORF) finder in the National Center for Biotechnology Information (NCBI)^2^ was used to identify the ORFs. SignalP-5.0 server was used to analyze the signal peptide of the amino acid sequence.^3^ ExPASy was used to calculate the theoretical isoelectric point (pI) and molecular weight (Mw) of Gal42.^4^ Conserved Domain Database (CDD) was used to improve the phylogenetic analysis. In addition, a BLAST algorithm program was performed to search similar sequences for Gal42. The ESPript^5^ and ClustalX program were used for multiple comparison analysis of Gal42 and BCA-β-Gal (PDB ID: 3TTS). MEGA 7.0 software was used to construct the evolutionary tree.

### Molecular Modeling

The three-dimensional (3D) structure of Gal42 was built by homology modeling using the SWISS MODEL.^6^ The crystalline structure of β-galactosidase from *Bacillus circulans* sp. *alkalophilus* (PDB ID: 3TTS) with sequence identity of 64.36% was chosen as the template. The Global Model Quality Estimation (GMQE) and Qualitative Model Energy Analysis (QMEAN) values for the homology model were calculated to evaluate the model quality and reliability. Pymol (version 2.4.1) was used to visualize the 3D structure, build organization graphics, and draw illustrations.

### Expression and Purification of Recombinant β-Galactosidase

*gal42* gene was synthesized by Synbio Technologies (Suzhou, China) and inserted into corresponding sites of plasmid pET-28a(+) between the recognition sites *Nco*I and *Xho*I. After the recombination, the plasmid was expressed in *E. coli* BL21 (DE3), which was cultured in Terrific Broth (TB) medium containing 30 μg/ml of kanamycin at 37°C until the optical density at 600 nm (OD_(600)_) reached 0.6–0.8. Afterward, the addition of 0.1 mM of isopropyl β-D-thiogalactoside (IPTG) was performed to induce the expression of the target protein at 20°C and 200 rpm for 36 h. The target β-galactosidase was purified and harvested in AKTA150 FPLC system using Ni-NTA Sepharose affinity column (5 ml His-Trap^TM^ High Performance, GE Healthcare, Madison, WI, United States). The supernatant that was obtained by the crude enzyme incubated in 50 mM of phosphate buffer (pH 7.6) was centrifuged for 10 min at 12,000 rpm. After sonication at ice condition (150 W, 2-s burst, and 2-s stop, 30 min), the obtained supernatant was loaded into the previously equilibrated affinity column. Then, the washing buffer (20 mM of imidazole, 20 mM of phosphate buffer, and 500 mM of NaCl, pH 7.6) was used to deplete proteins other than target proteins. The target protein was eluted by elution buffer (150 mM of imidazole, 20 mM of phosphate buffer, and 500 mM of NaCl, pH 7.6). Mw and purity of the enzymes were assayed by 10% sodium dodecyl sulfate–polyacrylamide gel electrophoresis (SDS-PAGE), and the protein concentration was determined by the bicinchoninic acid (BCA) protein assay kit (Beyotime Biotechnology, Shanghai, China). In brief, protein samples (2 μg) were resolved by 10% SDS-PAGE (the voltage of 75 V for concentrated glue and 120 V for separating glue), and Coomassie brilliant blue was used for staining.

### Activity Assay

The activity of β-galactosidase can be reflected by the production of ONP using ONPG as substrate. Briefly, 50 μl of diluted enzyme sample was mixed with 450 μl of 10 mM ONPG solution (20 mM of phosphate buffer, pH 7.0). Then, the reaction system was incubated at 40°C for 10 min and stopped by adding 500 μl of Na_2_CO_3_ (1 M). Furthermore, the released ONP was measured at 420 nm. Under the experimental conditions, one unit (U) of β-galactosidase activity was defined as the amount of enzyme required to release 1 μmol of ONP per minute (Li et al., 2019).

### Effect of Temperature, pH, Metal Ions, and Chelators on β-Galactosidase Activity

In order to determine the optimal temperature of the Gal42, substrate reactions for Gal42 and ONPG were carried out at different temperature ranging from 0 to 70°C. To determine the thermal stability of Gal42, the residual activity was measured after incubation at 0–70°C for 60 min. To examine the optimal pH, substrates for Gal42 and ONPG were carried out in Britton–Robinson buffers at pH 5.11–10.42. To determine the pH stability of Gal42, the residual enzyme activity was measured after Gal42 was cultured in buffers with different pH values at 4°C for 12 h. By monitoring the enzyme activity in the presence of different cations [using 1 mM of BaCl_2_, FeSO_4_, CaCl_2_, CuSO_4_, FeCl_3_, KCl, MnCl_2_, Al_2_(SO_4_)_3_, CoCl_2_, NaCl, (NH_4_)_2_SO_4_, NiCl, MgSO_4_, Li_2_SO_4_, and ZnSO_4_ in 20 mM of phosphate buffer] or chelating agents (1 mM of ethylenediamine tetraacetic acid (EDTA) and SDS), the effects of metal ions and chelating agents on Gal42 activity were determined as previously. Meanwhile, the effects of different concentrations of zinc ions (1, 5, and 10 mM) on optimal temperature and thermal stability were also detected according to the above conditions.

### Reaction Product Analysis

The reaction product of Gal42 in lactose and milk was determined by TLC. Briefly, 900 μl of lactose (5 mg/ml) and 100 μl of Gal42 were mixed and incubated under pH 8.0 at 40°C for 480 min. The reaction products were collected and boiled for 10 min at different time points (0, 1, 10, 30, 60, 360, and 480 min) to inactivate the enzyme. After that, 4 μl of sample from each time point was spotted in a horizon line on the silica gel TLC plate, and the plate was developed with a 3:2:5 (by vol.) mixture of glacial acetic acid, water, and 1-butanol. Later, after drying, the plate was treated with a 9:1 mixture of atomized ethanol and sulfuric acid. Heat the plate for 30 min at a temperature of 80°C, and the hydrolyzed products could be observed. The hydrolyzed products of lactose in drinking milk (4.2–5.0% lactose) (Inner Mongolia Mengniu Dairy Group Co., Ltd., Hohhot, China) was also observed by using the method described above. The diluted milk was mixed with enzymes, sampled at the same point, and then analyzed by TLC method, with galactose as the standard. Negative-ion electrospray ionization–mass spectrometry (ESI-MS) system (Thermo Fisher Scientific^TM^ Q Exactive^TM^ Hybrid Quadrupole-Orbitrap^TM^, Waltham, MA, United States) was employed to further investigate the composition of the reaction products. The instrument conditions were syringe pump injection-flow rate 50 μl/min, sheath gas 15 L/min, auxiliary gas 5 L/min, auxiliary gas temperature 150°C, capillary temperature 300°C, and S-lens voltage 50 V.

### Nucleotide Sequence Accession Numbers

The β-galactosidase gene (*gal42*) of strain *Bacillus* sp. BY02 was deposited in GenBank under accession number MW246571.

## Results and Discussion

### Isolation and Sequence Analysis

Thus far, several β-galactosidases have been purified and identified from bacteria (Mavromatis et al., 2010; Tian et al., 2013) and fungi (Rico-Díaz et al., 2017), including genus of *Bacillus* (Boon et al., 2000), *Bifidobacterium* (Hsu et al., 2007), and *Aspergillus* (Saqib et al., 2017). However, the productivity of β-galactosidases from natural microbial cells hardly meets the needs of industrial application due to the low yield (Park and Oh, 2010; Oliveira et al., 2011). Boosting the reaction efficiency of β-galactosidases is a precondition for achieving its industrial application. In a previous study, Becerra et al. (2001) has improved the partial secretion percentage of β-galactosidases in the culture medium through directing mutations at the N-terminus of the protein. Gutshall et al. (1995) reported that the β-galactosidase LacG isolated from *Arthrobacter* sp. B7 has a high specific activity. Thus far, the main industrial sources of β-galactosidase are *Aspergillus* sp. and *Kluyveromyces* (de Freitas et al., 2020).

In this study, the marine bacterium *Bacillus* sp. BY02 showed high intracellular β-galactosidase activity (19 U/ml) when grown in the 2216E medium containing 2% lactose. As shown in Supplementary Figure 1, it can produce β-galactosidase in the presence of lactose on 2216E X-Gal agar. The genome sequence analysis of *Bacillus* sp. BY02 showed that it contains a putative galactosidase-encoding gene, *gal42*, which consists of an ORF of 2,010 bp. The prepared β-galactosidase Gal42 contains 669 amino acid residues. The predicted Mw and theoretical pI of the expressed β-galactosidase were 78.38 kDa and 4.46, respectively.

**FIGURE 1:**
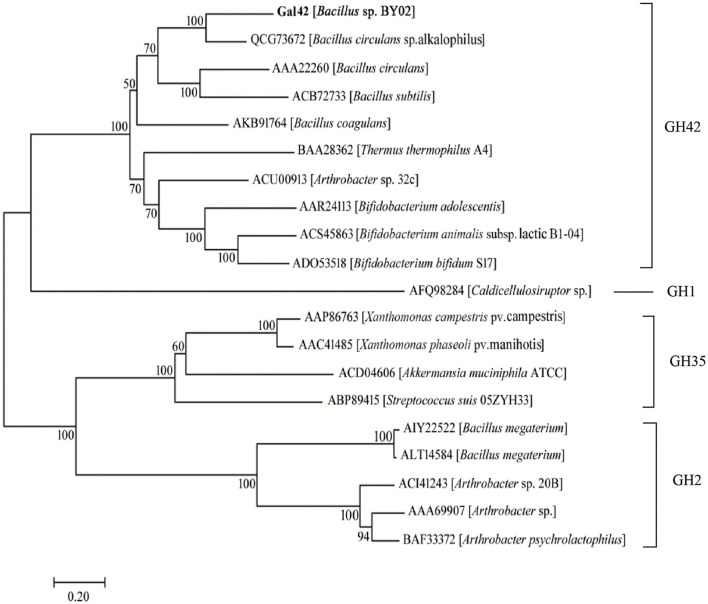
Evolutionary analysis of β-galactosidases. The phylogenetic tree of Gal42 and other β-galactosidases was constructed using the ClustalX program based the reference 16S rDNA sequences, which were collected from the National Center for Biotechnology Information (NCBI) database. The phylogenetic tree (1,000 bootstraps) was constructed using the MEGA 7.0 program *via* neighbor-joining method.

A phylogenetic tree was constructed containing Gal42 and other reported β-galactosidases in GH families 1, 2, 35, and 42 (Figure 1). In this neighbor-joining tree, Gal42 formed a monophyletic cluster with the β-galactosidase from GH42. In order to further explore the conserved and catalytic domains of Gal42, a multiple sequence alignment was established among Gal42 and other three β-galactosidase, Bca-β-gal from *Bacillus* (GenBank code: QCG73672), BI Gal42A from *Bifidobacterium animalis* subsp. (GenBank code: ACS45863), Bbg II from *Bifidobacterium bifidum* S17 (GenBank code: ADO53518), β-gal II from *Bifidobacterium adolescentis* (GenBank code: AAR2413), and A4-β-Gal from *Thermus thermophilus* A4 (GenBank code: BAA28362) (Supplementary Figure 2). According to multiple sequence alignment analysis, the acid/base catalyst site (Glu148) and the nucleophilic site (Glu305) conserved in the GH42 β-galactosidases were also present in the amino acid sequence of Gal42. The further BLAST analysis and comparison with the NCBI CDD clarified Gal42 as a GH42 enzyme, which showed the highest homology with the β-galactosidase from *Neobacillus bataviensis* (GenBank code: TWE08672, 80.45% identity) and the β-galactosidase from *Mesobacillus foraminis* (GenBank code: TCN25424, 79.10% identity). These results indicated that Gal42 is a new member of the GH42 β-galactosidase.

### Three-Dimensional Structure Analysis

**FIGURE 2:**
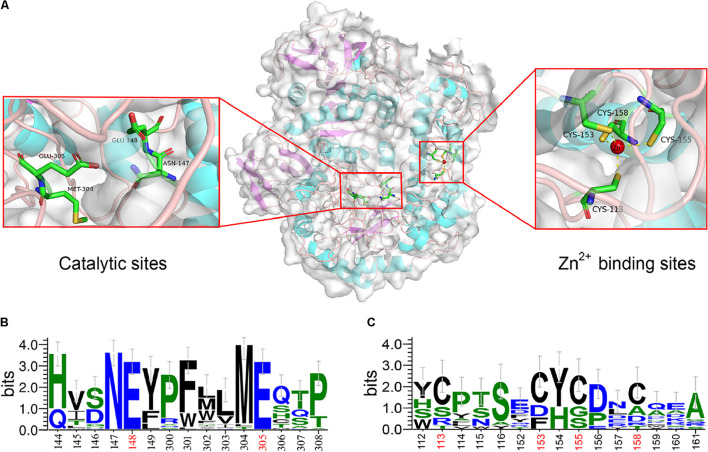
Sequence logo analysis and homology modeling of Gal42. **(A)** The key residues for substrate specificities in Gal42 are shown as dots mode. **(B)** Sequence logo analysis of Gal42 catalytic sites. **(C)** Sequence logo analysis of Gal42 metal binding sites.

The structural characterization indicated that the encoding β-galactosidase, Gal42, is a homotrimer in solution. As shown in Figure 2A, the 3D structure of Gal42 was constructed using SWISS-MODEL on the basis of homologs of known structure (Bca-β-gal, PDB ID: 3TTS). The GMQE and QMEAN values for the homology model were 0.87 and −3.05, respectively, indicating good model quality and high reliability. The homologous modeling result showed that Gal42 preserved the catalytic sites (Asn147, Glu148, Met304, and Glu305) and zinc binding sites of Cys cluster (Cys11, Cys153, Cys155, and Cys158). According to the sequence logo analysis (Figure 2B), two regions, “NEY” (Asn147-Glu148-Tyr149) and “ME” (Met304-Glu305), were conserved in the catalytic cave (Maksimainen et al., 2012). The catalytic residues Glu148 and Glu305 are highly conserved in all GH42 β-galactosidases, which play a role as an acid/base catalyst and a nucleophilic site, respectively (Barrangou et al., 2009). In addition, the Asn147 and Met304 preceding the two sites are also highly conserved. Tyr275, which is invariant in GH42, forms a hydrogen bond with the catalytic nucleophile Glu305, consistent with its proposed role as regulator. Trp313 provides aromatic stacking in GH42 and may modulate specificity by adopting different conformations (Hinz et al., 2004). The Zn^2+^ binding sites of Gal42 maintain its zinc coordination function in the Gal42 structure (Figure 2A). Meanwhile, as shown in Figure 2C, Cys cluster was not conserved in GH42 β-galactosidases, which illustrated that possession of metal binding sites was not a commonality in β-galactosidases.

### Purification and Biochemical Characterization

**FIGURE 3:**
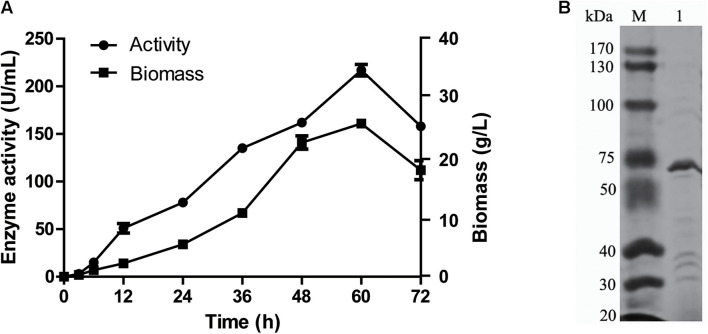
Expression and purification of recombinant Gal42. **(A)** Time curve of Gal42 activity secreted into culture medium. **(B)** Sodium dodecyl sulfate–polyacrylamide gel electrophoresis (SDS-PAGE) analysis of the Gal42. The protein samples were separated by 10% SDS-PAGE gel and stained with Coomassie Blue G-250. Lane M, protein marker; Lane 1, the purified Gal42.

*gal42* gene was cloned and overexpressed in *E. coli* BL21(DE3) through the pET28a(+) plasmid. The formed *E. coli* BL21-pET28a-gal42 strain was cultured in Luria Bertani (LB) broth for proliferation and then in TB medium for enzyme production with IPTG. As shown in Figure 3A, the β-galactosidases activity of purified Gal42 was up to 217 U/ml, with biomass of 27 g/L at 60 h. The Mw of the purified Gal42 was analyzed by SDS-PAGE and showed to be about 74 kDa with a dominating band (Figure 3B), which is consistent with its theoretical Mw (78.38 kDa).

**FIGURE 4:**
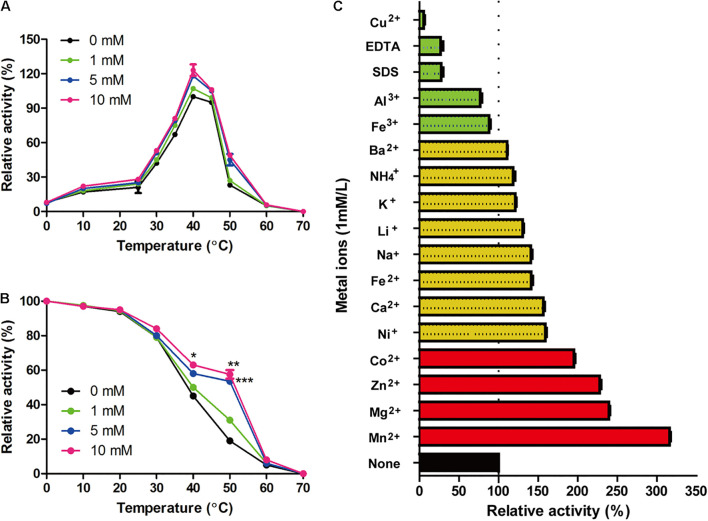
Effect of zinc ions and other metal ions on enzymatic activity of Gal42. **(A)** Effect of zinc ions and temperature on the activity of Gal42. **(B)** Effect of zinc ions on thermo-stability of Gal42. **(C)** Effects of metal ions, EDTA, and SDS on the activity of Gal42. Activity without addition of chemicals was defined as 100%. The values are the mean values ± standard deviations of three experiments in three replicates. *p < 0.05; **p < 0.01; and ***p < 0.001.

As shown in Figure 4A, the optimal reaction temperature of the purified Gal42 was 40°C. In the thermal stability assays, Gal42 was kept stable at 0–30°C (Figure 4B). The enzyme remained approximately 50% activity even after 40°C incubation. The optimal pH of Gal42 in Britton–Robinson buffers was determined to be 7.44 (Supplementary Figure 3A), which is similar to that of other GH42 β-galactosidases (Hu et al., 2007; Hildebrandt et al., 2009). Additionally, after a 12-h pretreatment in Britton–Robinson buffers at pH 5.11–10.42, the enzyme remained stable at pH 6.0–8.5, with an activity exceeding 60% (Supplementary Figure 3B). Especially, the residual activity of Gal42 was nearly 100% of its maximum activity at pH range 7.0–8.0. Compared with a pH-stable β-galactosidase BgaL from *Paracoccus* sp. 32d (Wierzbicka-Woś et al., 2011), Gal42 has shown wider pH-stability range. Considering the neutral pH of milk, β-galactosidase Gal42 was easily utilized and suitable in producing lactose-free milk with its properties mentioned above.

### Effect of Zinc Ions on Biochemical Characterization

β-Galactosidase Gal42 contained a zinc binding site of the Cys cluster. Herein, the effect of zinc ions on biochemical characterization of Gal42 was determined (Figure 4). In the presence of Zn^2+^, the enzyme activity of Gal42 was significantly increased at the temperature of 10–60°C. At 40°C, the enzyme activity of Gal42 increased to 119 and 126% with 5 and 10 mM of zinc ions, respectively. However, the optimal temperature of the Gal42 was not affected by Zn^2+^ (Figure 4A). In the presence of 1 mM of Zn^2+^, the thermo-stability of Gal42 was slightly increased; and in the presence of 5 mM of Zn^2+^, the thermo-stability of Gal42 was significantly increased on 40 and 50°C (*p* < 0.001). Meanwhile, by increasing the concentration of Zn^2+^ to 10 mM, the activity of Gal42 was not significantly increased anymore (Figure 4B). As shown in Figure 4C, Cu^2+^, EDTA, and SDS showed significant inactivation effects on the activity of Gal42. The activity of Gal42 can be activated by Li^+^, Ba^2+^, Fe^2+^, Ca^2+^, K^+,^ NH_4_^+^, Ni^+^, and Na^+^; while it was slightly inhibited by Fe^3+^ and Al^3+^. More interestingly, Gal42 can be activated not only by Zn^2+^ but also by divalent metal ions (Co^2+^, Mn^2+^, and Mg^2+^). As shown in previous studies, the metal ions of Mn^2+^, Mg^2+^, Zn^2+^, and Ni^+^ could greatly inhibit the activity of a β-galactosidase from *Alteromonas* sp. ML52 over 32.4% (Sun et al., 2018). The results further showed that zinc ion plays an important role in Gal42, and it is a novel enzyme with different characteristics from other enzymes belonging to GH42.

The presence of ions often affects the activity and stability of β-galactosidase. The β-galactosidase obtained from bacterial strain *Erwinia* sp. E602 (Xia et al., 2018) was also obviously inactivated by Cu^2+^ with about 5% activity. Mg^2+^ and Na^+^ are both required to achieve the maximal activity of the β-galactosidase (Juers et al., 2001). The active site of Mg^2+^ can be substituted by Mn^2+^ without many differences in activity. In this study, Gal42 showed obviously enhancing activity when Mn^2+^ and Mg^2+^ presented, and the presence of Na ^+^ also increases the activity of Gal42. Mn^2+^ and Mg^2+^ are beneficial to many reported β-galactosidase activities, such as enzymes from *Pediococcus pentosaceus* and *Pediococcus acidilactici* (Lee et al., 2017; Chanalia et al., 2018). In the aspect of structural analysis, Mg^2+^ binds to each subunit and has the largest influence on the nearest substrate binding site. It also can modulate the chemistry of active site components (Lo et al., 2010). Another important role of Mg^2+^ is that it can stabilize a mobile loop at the active site through interacting with Glu residue (Sutendra et al., 2007). What is more interesting is, in the presence of Zn^2+^, the activity and thermo-stability of Gal42 were significantly increased. There are three types Zn^2+^ binding sites in proteins: catalytic, co-catalytic, and structural. Among them, the Zn^2+^ binding is coordinated by four amino acid side chains, including either four cysteines or two histidines in combination with two cysteines (Shumilina et al., 2014). Maksimainen et al. (2012) have demonstrated that Zn^2+^ does not play a role in the catalytic cycle in Bca-β-gal; however, the binding of Zn^2+^ stabilized the structure of the catalytic domain. In the same sense, our experimental results showed that the activity and thermal stability of Gal42 were improved in the presence of zinc ion (Figure 4). The improved thermo-stability of Gal42 with the presence of Zn^2+^ may be due to the greatly increased stability of Zn^2+^ binding site (Cys113, Cys153, Cys155, and Cys158) (Figure 2).

### Analysis of Hydrolysates From Lactose and Milk

**FIGURE 5:**
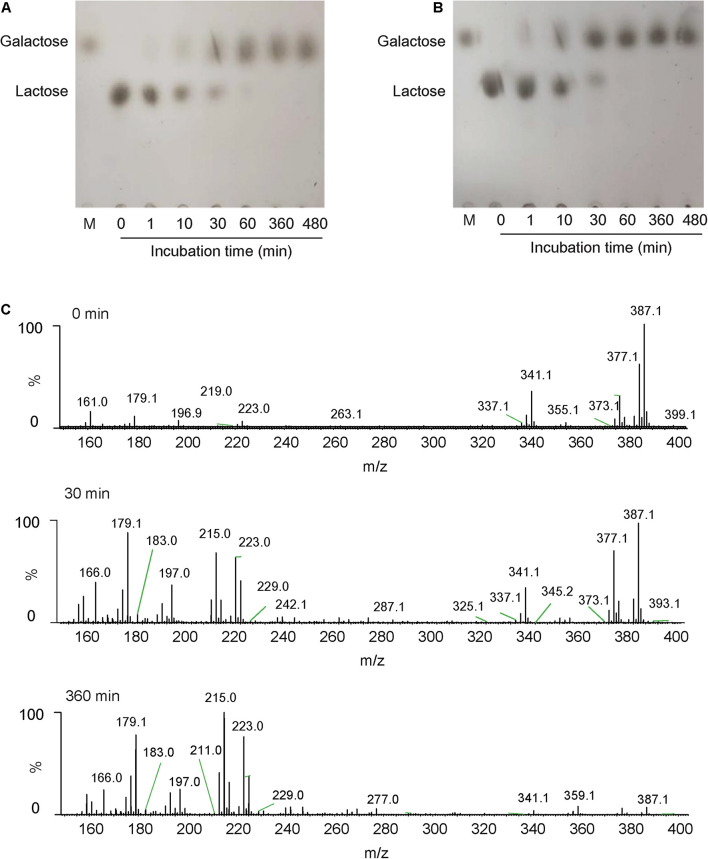
The analysis of action patterns and reaction products for Gal42. **(A)** Thin-layer chromatography (TLC) analysis of Gal42-hydrolyzed lactose. **(B)** TLC analysis of Gal42-hydrolyzed milk. Lane M, standard galactose. **(C)** Electrospray ionization–mass spectrometry (ESI-MS) analysis of reaction products for Gal42 after 0-, 30-, and 360-min incubation.

In this study, lactose and milk were used as the substrates, and the reaction was carried out for a certain time under the catalysis of Gal42 to detect and analyze the decomposition of lactose. TLC was used to analyze its reaction product. As shown in Figure 5A, monosaccharides are formed by hydrolysis of lactose during the reaction. After 360 min, lactose had been fully hydrolyzed into monosaccharides. The reaction products were further identified by negative ESI-MS. As shown in Figure 5C, the main peak at 341.1 and 377.1 *m/z* corresponded to lactose, and 179.1 and 223.0 *m/z* corresponded to galactose. These results indicated that the lactose almost fully hydrolyzed into galactose within 360 min.

According to the properties of Gal42, the enzyme activity can be relatively stable in a certain range of pH (covers the pH range of milk). Therefore, it was completely feasible to use Gal42 to hydrolyze lactose in milk in industrial application. In this study, it could be observed that the lactose content in milk can decreased with the enzyme reaction of Gal42. The reaction was completed within 60 min, and all lactose was decomposed into monomers (Figure 5B). In conclusion, this characteristic indicates that Gal42 may have a good application prospect in dairy industry.

## Conclusion

Herein, a new GH42 β-galactosidase-encoding gene was cloned and overexpressed. The recombinant β-galactosidase showed high yield, pH stability, and thermal stability. The “Cys-Zn” motif played important roles in both structural stability and catalytic function. With the present Zn^2+^, thermo-stability of Gal42 was improved. Furthermore, Gal42 showed high lactose hydrolysis activity, which enables the enzyme to effectively hydrolyze the lactose in milk. These biochemical properties make Gal42 a potential candidate for food technology applications.

## Data Availability Statement

The datasets presented in this study can be found in online repositories. The names of the repository/repositories and accession number(s) can be found in the article/Supplementary Material.

## Author Contributions

ShL and JH designed the experiments. ZZ, NH, QH, and ShL analyzed the data and wrote the main manuscript text. RX and NH carried out three-dimensional modeling and analysis. All authors have read and agreed to the published version of the manuscript.

## Conflict of Interest

The authors declare that the research was conducted in the absence of any commercial or financial relationships that could be construed as a potential conflict of interest.

## Publisher’s Note

All claims expressed in this article are solely those of the authors and do not necessarily represent those of their affiliated organizations, or those of the publisher, the editors and the reviewers. Any product that may be evaluated in this article, or claim that may be made by its manufacturer, is not guaranteed or endorsed by the publisher.

## Footnotes

1
www.cazy.org

2
https://www.ncbi.nlm.nih.gov/orffinder/

3
http://www.cbs.dtu.dk/services/SignalP/

4
https://web.expasy.org/cgibin/protparam/protparam

5
http://espript.ibcp.fr/ESPript/ESPript/

6
https://swissmodel.expasy.org/

## Supplementary Material

Supplementary Figure 1 Effects of glucose and lactose on the expression of β-galactosidase of *Bacillus* sp. BY02. *E. coli* strain BL21(DE3) was used as a control. **(A)** β-galactosidase selective medium with 2% glucose. **(B)** β-galactosidase selective medium with 2% lactose. **(C)** β-galactosidase selective medium with 2% glucose and lactose.

Supplementary Figure 2 Sequence comparison of Gal42 with related β-galactosidase from GH family 42 and a β-galactosidase (PDB: 3TTS) from *Bacillus circulans* sp. alkalophilus. Identical residues are shaded in red. β-Gal II from *Bifidobacterium adolescentis* (Genbank code: AAR24113); Bca-β-gal from Bacillus *circulans* sp. alkalophilus (Genbank code: QCG73672); Bbg II from *Bifidobacterium bifidum* S17 (Genbank code: ADO53518); BI Gal42a from *Bifidobacterium animalis* subsp. (Genbank code: ACS45863) and A4-β-Gal from *Thermus thermophilus* A4 (Genbank code: BAA28362). The catalytic sites and metal binding sites are labeled in red and black pentagrams, respectively. The black arrows domain structure **(A–C)**.

Supplementary Figure 3 Effect of pH on Gal42 enzymatic activity. **(A)** Effect of pH on the activity of Gal42 in Britton-Robinson buffers (pH 5.11–10.42); **(B)** pH-stability of Gal42. Gal42 pH stability was incubated at 4°C for 12 h in Britton-Robinson buffers (pH 5.11–10.42), and then residual activity was assayed under normal assay conditions. Values are the mean values ± standard deviations of three experiments in three replicates.
