# Supplementary material for: Characterization and Application of a New β-Galactosidase Gal42 From Marine Bacterium Bacillus sp. BY02
Source: Front Microbiol. 2021 Oct 25;12:742300. doi: 10.3389/fmicb.2021.742300 (PMC8573354; doi:10.3389/fmicb.2021.742300)
Supplement: Supplementary Figure 1 — Effects of glucose and lactose on the expression of β-galactosidase of Bacillus sp. BY02. E. coli strain BL21(DE3) was used as a control. (A) β-galactosidase selective medium with 2% glucose. (B) β-galactosidase selective medium with 2% lactose. (C) β-galactosidase selective medium with 2% glucose and lactose. [file Data_Sheet_1.PDF]

Figure S1

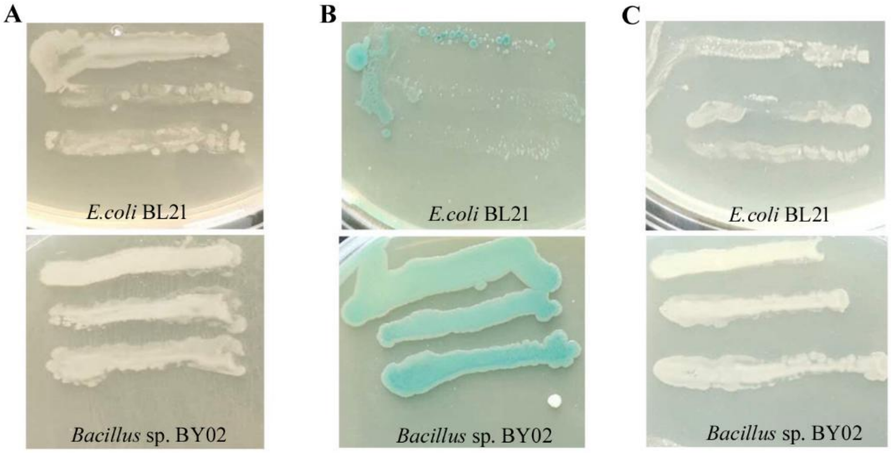

Figure S1. Effects of glucose and lactose on the expression of β-galactosidase of *Bacillus* sp. BY02. *E. coli* strain BL21(DE3) was used as a control. (**A**) β-galactosidase selective medium with 2% glucose. (**B**) β-galactosidase selective medium with 2% lactose. (**C**) β-galactosidase selective medium with 2% glucose and lactose.

Figure S2

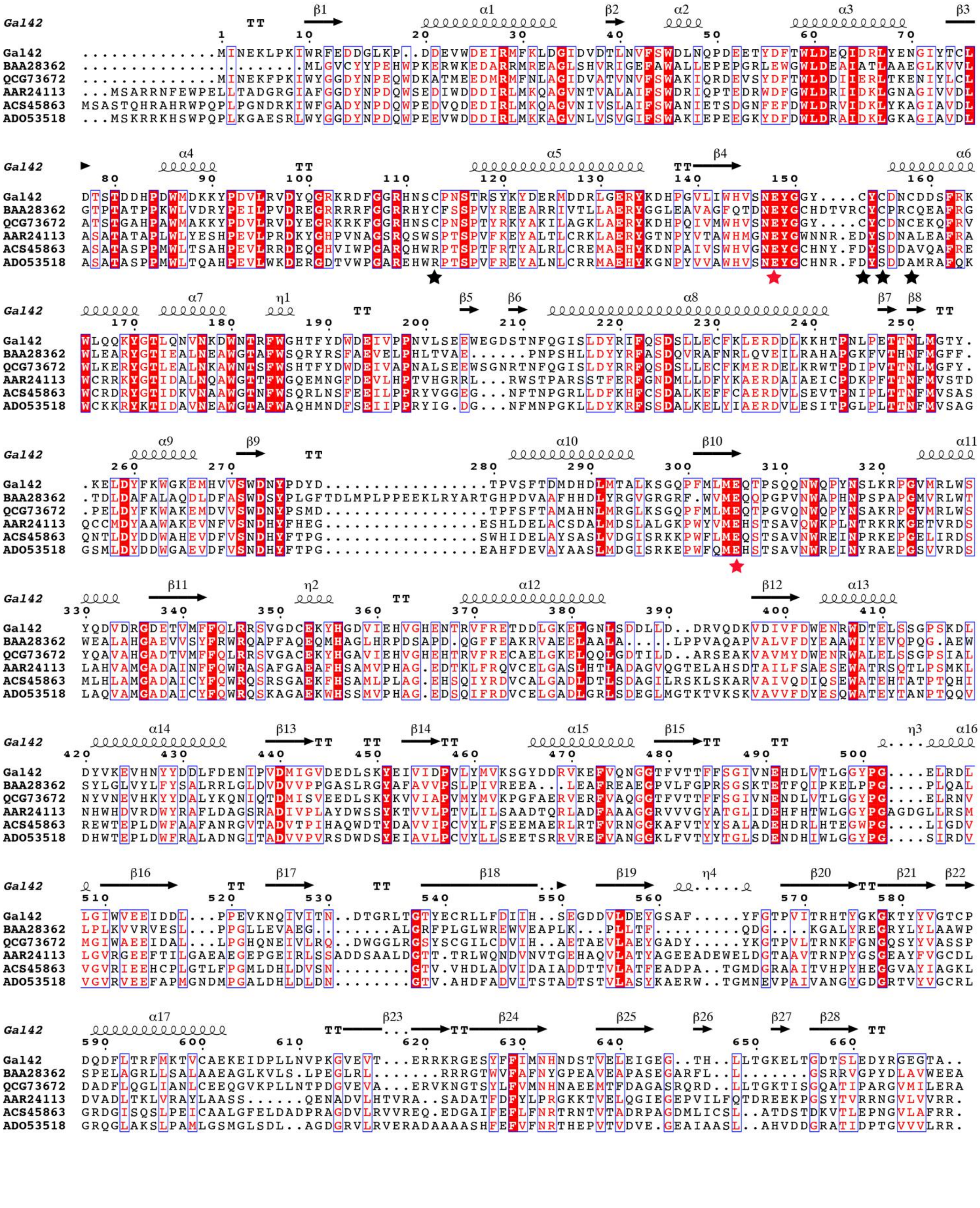

Figure S2. Sequence comparison of Gal42 with related β-galactosidase from GH family 42 and a β-galactosidase (PDB: 3TTS) from *Bacillus circulans* sp. alkalophilus. Identical residues are shaded in

red. β-Gal II from *Bifidobacterium adolescentis* (Genbank code: AAR24113); Bca-β-gal from Bacillus *circulans* sp. alkalophilus (Genbank code: QCG73672); Bbg II from *Bifidobacterium bifidum* S17 (Genbank code: ADO53518); BI Gal42a from *Bifidobacterium animalis* subsp. (Genbank code: ACS45863) and A4-β-Gal from *Thermus thermophilus* A4 (Genbank code: BAA28362). The catalytic sites and metal binding sites are labeled in red and black pentagrams, respectively. The black arrows domain structure (**A**, **B** and **C**).

Figure S3

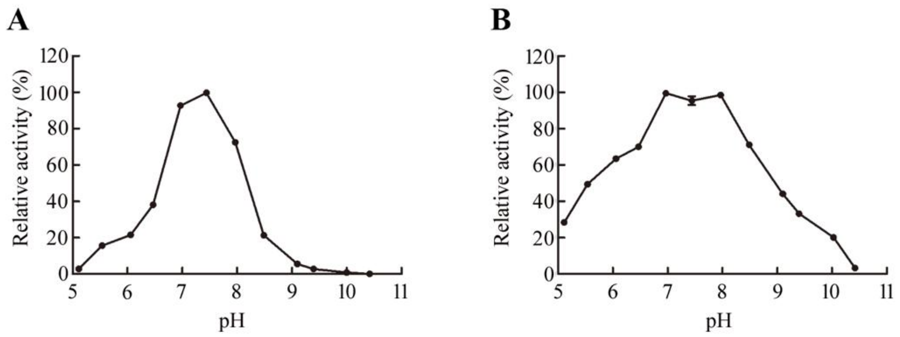

Figure S3. Effect of pH on Gal42 enzymatic activity. (**A**) Effect of pH on the activity of Gal42 in Britton-Robinson buffers (pH 5.11-10.42); (**B**) pH-stability of Gal42. Gal42 pH stability was incubated at 4 °C for 12 h in Britton-Robinson buffers (pH 5.11-10.42), and then residual activity was assayed under normal assay conditions. Values are the mean values ± standard deviations of three experiments in three replicates.
